# The effect of nicotinamide adenine dinucleotide phosphate oxidase 4 on migration and invasion of fibroblast-like synoviocytes in rheumatoid arthritis

**DOI:** 10.1186/s13075-020-02204-0

**Published:** 2020-05-15

**Authors:** Ha-Reum Lee, Su-Jin Yoo, Jinhyun Kim, In Seol Yoo, Chan Keol Park, Seong Wook Kang

**Affiliations:** 1 Research Institute for Medical Sciences, Chungnam National University School of Medicine, 266 Munhwaro, Daejeon, Republic of Korea; 2 Division of Rheumatology, Department of Internal Medicine, Chungnam National University Hospital, 282 Munhwaro, Daejeon, 35015 Republic of Korea

**Keywords:** Rheumatoid arthritis, Osteoarthritis, Fibroblast-like synoviocytes, Reactive oxygen species, NADPH, NOX4, VCAM1, VEGF

## Abstract

**Background:**

Reactive oxygen species (ROS) regulate the migration and invasion of fibroblast-like synoviocytes (FLS), which are key effector cells in rheumatoid arthritis (RA) pathogenesis. Nicotinamide adenine dinucleotide phosphate oxidase 4 (NOX4) induces ROS generation and, consequently, enhances cell migration. Despite the important interrelationship between RA, FLS, and ROS, the effect of NOX4 on RA pathogenesis remains unclear.

**Methods:**

FLS isolated from RA (*n* = 5) and osteoarthritis (OA, *n* = 5) patients were stimulated with recombinant interleukin 17 (IL-17; 10 ng/ml) and tumor necrosis factor alpha (TNF-α; 10 ng/ml) for 1 h. Cell migration, invasion, adhesion molecule expression, vascular endothelial growth factor (VEGF) secretion, and ROS expression were examined. The mRNA and protein levels of NOX4 were analyzed by RT-qPCR and western blotting, respectively. The NOX4 inhibitor GLX351322 and NOX4 siRNA were used to inhibit NOX4 to probe the effect of NOX4 on these cellular processes.

**Results:**

Migration of RA FLS was increased 2.48-fold after stimulation with IL-17 and TNF-α, while no difference was observed for OA FLS. ROS expression increased in parallel with invasiveness of FLS following cytokine stimulation. When the expression of *NOX* was examined, *NOX4* was significantly increased by 9.73-fold in RA FLS compared to unstimulated FLS. Following NOX4 inhibition, cytokine-induced vascular cell adhesion molecule 1 (VCAM1), VEGF, and migration and invasion capacity of RA FLS were markedly decreased to unstimulated levels.

**Conclusion:**

NOX4 is a key contributor to cytokine-enhanced migration and invasion via modulation of ROS, VCAM1, and VEGF in RA FLS.

## Key message

- NOX4 mediated a differential response to IL-17 and TNF-α stimulation in RA FLS compared to OA FLS
- *NOX4* was increased by cytokine exposure and was required for RA FLS migration and invasion via increased expression of VCAM1 and VEGF

## Background

Rheumatoid arthritis (RA) is a systemic autoimmune disease that is characterized by joint inflammation and bone destruction [1, 2]. During RA pathogenesis, there is excessive immune cell infiltration into synovial joints, stimulating other immune cells, endothelial cells, and fibroblast-like synoviocytes (FLS) [3]. Activated FLS can cause synovial hyperplasia in the joint, attach to and invade the cartilage surface, cause destruction of the cartilage and bone, and activate immune responses [4]. Although migration and invasion of FLS play important roles in the initiation and development of RA, these processes are induced by diverse factors through complex pathways [5].

Reactive oxygen species (ROS), which are more abundant in RA patients than in controls, are considered to be a significant contributor to RA pathogenesis [6, 7]. ROS act as signaling molecules to modulate cell migration, proliferation, survival, and homeostasis [8]. Although a certain amount of ROS is essential for cell protection, excessive oxidative stress contributes to inflammation, cancer, aging, and autoimmune diseases. Most intracellular ROS are generated from mitochondria and catalyzed by the enzyme complex nicotinamide adenine dinucleotide phosphate (NADPH) oxidase (NOX) [9, 10]. There are seven of NOX proteins: NOX1–5 and DUOX1–2. Notably, NOX4 induces ROS generation and cell migration in human lung endothelial cells [11]. Recently published data suggest that the proangiogenic factor leptin triggers the migration and invasion of RA FLS via elevated ROS generation [12]; however, the specific mechanism of NOX4 in FLS invasion remains to be fully characterized in RA.

In this study, we induced pro-inflammatory conditions in FLS using recombinant interleukin 17 (IL-17) and tumor necrosis factor alpha (TNF-α) treatment, as these cytokines are critical in RA pathogenesis rather than OA [13, 14]. The pro-inflammatory cytokine IL-17 is highly expressed in RA synovium and directly stimulates FLS activation [15] while TNF-α inhibition is an effective universal therapy option for RA treatment. Because active migratory phenotype and strong cartilage invasiveness are unique characters of RA FLS [16], OA FLS were used as control. Actually, the primary faulty of OA is caused by cartilage rather than synovium in clinical outcome [17]. OA FLS have been widely used for comparison with RA FLS. Here, we investigated the effects of IL-17 and TNF-α on migration and invasion of FLS isolated from patients with RA and OA and further explored the effects of NOX4 inhibition in the pathogenesis of RA.

## Methods

### Human subjects and ethics statement

Synovial tissues were obtained from 5 female RA patients (average age 61.6 ± 1.67 years) and 5 female OA patients (average age 64.8 ± 16.10 years) who were undergoing synovectomy or joint replacement. The diagnosis of RA conformed to the College of Rheumatology (ACR)/European League Against Rheumatism (EULAR) 2010 classification criteria [18]. After removing fat and fibrous tissues, the synovium was cut into small pieces and incubated with 0.1% collagenase (Sigma-Aldrich) in Dulbecco’s modified Eagle’s medium (DMEM) at 37 °C for 3 h. Cells were cultured in DMEM supplemented with 10% fetal bovine serum (FBS, Gibco) and maintained in a 5% CO_2_ incubator at 37 °C. FLS were used for experiments after four to six passages. This study was performed according to the recommendations of the Declaration of Helsinki and approved by the Institutional Review Board of Chungnam National University Hospital (CNUH 2015-10-052).

### Transwell migration and Matrigel invasion assay

FLS were cultured in serum-free media for 5 h. Following pre-incubation with or without NOX4 inhibitor (GLX351322, MedKoo Biosciences) for 1 h, RA FLS were stimulated with recombinant IL-17 (10 ng/ml, Peprotech) and TNF-α (10 ng/ml, Peprotech) for 1 h. For the transwell migration assay, cells were centrifuged and loaded onto transwell filters with an 8-μm pore (Corning) positioned on top of the migration chamber for 23 h. DMEM containing 10% FBS was transferred to the bottom chamber of the transwell plate as a chemoattractant. For the invasion assay, transwell filters were pre-incubated with Matrigel (Corning) at 37 °C for 1 h. Then, transwells were incubated at 37 °C for 3 days, fixed with 100% methanol, and stained with crystal violet (Sigma-Aldrich). Non-migrating cells on the top membrane surface were removed by washing with PBS and cotton swabs. Invaded cells were counted in five random fields per sample under an inverted microscope (magnification, × 100; 0.55 numerical aperture dry objective; scale bar, 100 μm; Olympus). For quantification, the crystal violet dye was eluted with 0.1% sodium dodecyl sulfate (SDS) and quantitated using a Sunrise absorbance reader (Tecan) at 595 nm.

### Flow cytometric analysis

To analyze the intrinsic surface expressions of IL-17 or TNF receptors, FLS were stained using APC-conjugated anti-human CD217 (IL-17Ra; Invitrogen), BV421 anti-human CD120b (TNF Receptor Type II; BD Biosciences), and mouse anti-human CD120a (TNF Receptor Type I; BD Biosciences) with FITC-conjugated anti-mouse IgG secondary antibody (BD Biosciences).

FLS cultures were incubated with serum-free media for 5 h and then stimulated with recombinant IL-17 and TNF-α for 1 h. Culture media was changed, and the cells were incubated with FITC-conjugated anti-human vascular cell adhesion protein 1 (VCAM1; BD Biosciences), PE-Cy™5-conjugated anti-human intercellular adhesion molecule 1 (ICAM1; BD Biosciences), and PE-Cy™7-conjugated anti-human neural cell adhesion molecule 1 (NCAM1; BD Biosciences). To detect ROS levels, cells were stained with MitoSOX™ Red mitochondrial superoxide indicator (Invitrogen) according to the manufacturer’s instructions. Cells were analyzed with a FACSCantoII flow cytometer (BD Biosciences), and data were processed with FlowJo software (Tree Star).

### Wound migration assay

When FLS cultures were approximately 90% confluent, cells were incubated with serum-free media for 5 h. FLS monolayers were wounded with pipette tips and treated with recombinant IL-17 and TNF-α for 1 h. Wound closure was monitored and photographed at 0 and 24 h with a Olympus inverted microscope (magnification, × 100; 0.55 numerical aperture dry objective; scale bar, 100 μm). To quantify the migrated cells, pictures of the initial wounded monolayers were compared with the corresponding pictures of cells at the end of the incubation.

### Enzyme-linked immunosorbent assay (ELISA)

VEGF concentrations were measured using ELISA kits for human VEGF (R&D Systems) according to the manufacturers’ instructions. VEGF levels were estimated by interpolation from a standard curve generated using a Sunrise absorbance reader (Tecan) at 450 nm.

### Real-time PCR and RT-PCR

Total RNA was extracted using TRI Reagent (Molecular Research Center), according to the manufacturer’s instructions. Extracted RNA was used in reverse transcription reactions with ReverTra Ace® qPCR RT Master Mix (TOYOBO) according to the manufacturer’s instructions. SYBR® Green Realtime PCR Master Mix (TOYOBO) was used for real-time PCR analysis of cDNA according to the manufacturer’s instructions. The primers were synthesized by Bioneer (see Table 1 for primer sequences). Thermal cycling conditions were as follows: initial denaturation at 95 °C for 5 min, 40 cycles of 95 °C for 10 s, 60 °C for 15 s, and 72 °C for 20 s. A melting step was performed by raising the temperature from 72 °C to 95 °C after the last cycle. Thermal cycling was conducted on a CFX Connect Real-Time PCR Detection System machine (Bio-Rad Laboratories). The target gene expression levels are shown as a ratio in comparison with the levels of glyceraldehyde 3-phosphate dehydrogenase (GAPDH) in in the same sample via calculation of the cycle threshold (Ct) value. The relative expression levels of target genes were calculated by the 2^−ΔΔCT^ relative quantification method.

**Table 1:** Primers used for PCR

	Sense primer	Antisense primer
***NOX1***	**GTTTTACCGCTCCCAGCAGAA**	**GGATGCCATTCCAGGAGAGAG**
***NOX2***	**CAAGATGCGTGGAAACTACCTAAGAT**	**TCCCTGCTCCCACTAACATCA**
***NOX3***	**ACCGTGGAGGAGGCAATTAGACAA**	**CAGGTTGAAGAAATGCGCCACGAT**
***NOX4***	**CTCAGCGGAATCAATCAGCTGTG**	**AGAGGAACACGACAATCAGCCTTAG**
***NOX5***	**ATCAAGCGGCCCCCTTTTTTTCAC**	**CTCATTGTCACACTCCTCGACAGC**
***DUOX1***	**TTCACGCAGCTCTGTGTCAA**	**AGGGACAGATCATATCCTGGCT**
**p22phox(*CYBA*)**	**CGCTGGCGTCCGCCTGATCCTCA**	**ACGCACAGCCGCCAGTAGGTAGAT**
**p67phox(*NCF2*)**	**ATCAGCCTCTGGAATGAAGGGG**	**GCAGCCAATGTTGAAGCAAATCC**
***GAPDH***	**CACATGGCCTCCAAGGAGTAA**	**TGAGGGTCTCTCTCTTCCTCTTGT**

For RT-PCR, the synthesized cDNA was mixed with AccuPower® RT PreMix (Bioneer) and 10 pmol of each specific PCR primer following the manufacturer’s protocol. Amplified products were separated on 1% agarose gels, stained with Midori green advance (NIPPON Genetics), and photographed under UV illumination using a GelDoc system (Bio-Rad Laboratories).

### Western blot analysis

Cells were ruptured on ice using a RIPA lysis kit (ATTO Corporation), lysates were clarified by centrifugation, and samples were analyzed by sodium dodecyl sulfate-polyacrylamide gel electrophoresis (SDS-PAGE). Proteins were transferred onto polyvinylidene (PVDF) membranes (Bio-Rad), which were then incubated with antibodies against NOX4 (1/1000 dilution; Abcam), β-actin (1/2000 dilution; Sigma-Aldrich), PI3Kδ (1/1000 dilution; Cell Signaling Technology), and GAPDH (1/3000 dilution; Cell Signaling Technology) overnight at 4 °C. After washing with PBS-T, membranes were stained with peroxidase-conjugated goat anti-rabbit IgG (Abcam) or peroxidase-conjugated rabbit anti-mouse IgG (Abcam). Target proteins were then detected using the chemiluminescent HRP Substrate (Thermo Fisher Scientific).

### siRNA transfection

Specific siRNA targeting NOX4 was purchased from Santa Cruz Biotechnology (sc-41586). Cells were transfected with lipofectamine transfection reagent (Invitrogen) and the indicated siRNA duplex targeting constructs. After incubation for 24 h, downregulation of target expression was evaluated by RT-PCR and western blot analysis.

### Statistical analysis

Statistical analysis was performed using the paired Student’s *t* test in SPSS 18.0*. P* values < 0.05 were considered statistically significant.

## Results

### Increased migration and invasion in RA FLS

**Fig. 1:**
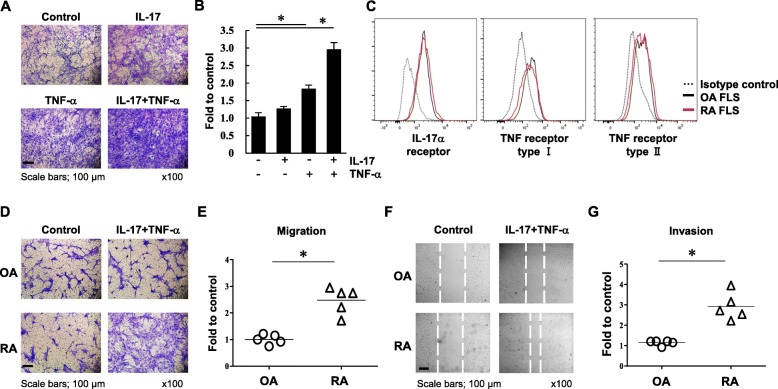
IL-17 and TNF-α stimulation promotes migration and invasion in RA FLS but not OA FLS. **a** Human FLS from donors with RA were incubated with or without IL-17 (10 ng/ml) and TNF-α (10 ng/ml) for 1 h. Cell migration was measured using a transwell chamber after 23 h and visualized with crystal violet staining. **b** Following solubilization, the crystal violet dye was quantitated. Data represent the fold change of the optical density of crystal violet-stained cells stimulated with IL-17 and TNF-α compared to unstimulated control cells. **c** To analyze the intrinsic surface expressions of IL-17 or TNF receptors, FLS were stained using APC-conjugated anti-human CD217 (IL-17Ra), BV421 anti-human CD120b (TNF receptor type II), and mouse anti-human CD120a (TNF receptor type I) with FITC-conjugated anti-mouse IgG secondary antibody. The isotype control is represented by the dotted line. OA FLS were indicated by the black line and RA FLS were represented by the red line. These were analyzed by flow cytometry. **d** After RA or OA FLS were stimulated with or without IL-17 (10 ng/ml) and TNF-α (10 ng/ml) for 1 h, cell migration was analyzed using a transwell chamber after 23 h. **e** The migrated cells were solubilized and quantitated. **f** Following scratch assay, the migrated cells were photographed. **g** Cell invasion was evaluated using Matrigel-coated transwell chambers after 3 days. Each symbol represents an individual donor, and the bar represents the mean. Data represent one experiment, which was performed in triplicate with similar results. *p < 0.05. Scale bar, 100 μm. Magnification is × 100

Migration of activated FLS mediates bone damage in RA progression via invasion through the cartilage [19]. We therefore investigated whether RA FLS have increased cell migration in response to stimulation with IL-17 and TNF-α. Following treatment with IL-17 and TNF-α for 1 h, migration of FLS from patients with RA and OA was examined using a transwell chamber assay. When RA FLS were stimulated with or without IL-17 or TNF-α, cell migration was powerfully enhanced by IL-17 and TNF-α together (Fig. 1a, b). To exclude the intrinsic difference in levels of cytokine receptors between RA and OA FLS, we evaluated the expression of cytokine receptors on FLS. There was no difference in the expression of IL-17 and TNF receptors between RA and OA (Fig. 1c). Cell migration was increased by 2.48-fold in RA FLS stimulated with cytokines compared to those without stimulation (2.48 ± 0.50), while cell migration did not differ for OA FLS in response to cytokine stimulation (1.01 ± 0.18; Fig. 1d, e). This differential migration in response to IL-17 and TNF-α was confirmed using a scratch assay in which cell movement into the wounded area was markedly increased in RA compared to OA cultures (Fig. 1f). When cell invasion was analyzed using a Matrigel-coated transwell membrane, IL-17 and TNF-α stimulation significantly enhanced the invasion ability of RA FLS compared to unstimulated RA FLS controls (2.92 ± 0.67, Fig. 1g). Invasion by OA FLS were not changed following cytokine treatment (1.14 ± 0.12). These data indicate that RA FLS have increased cell migration and invasion in response to IL-17 and TNF-α stimulation than OA FLS.

### Higher cytokine-induced expression of VCAM1, VEGF, and ROS in RA FLS than in OA FLS

**Fig. 2:**
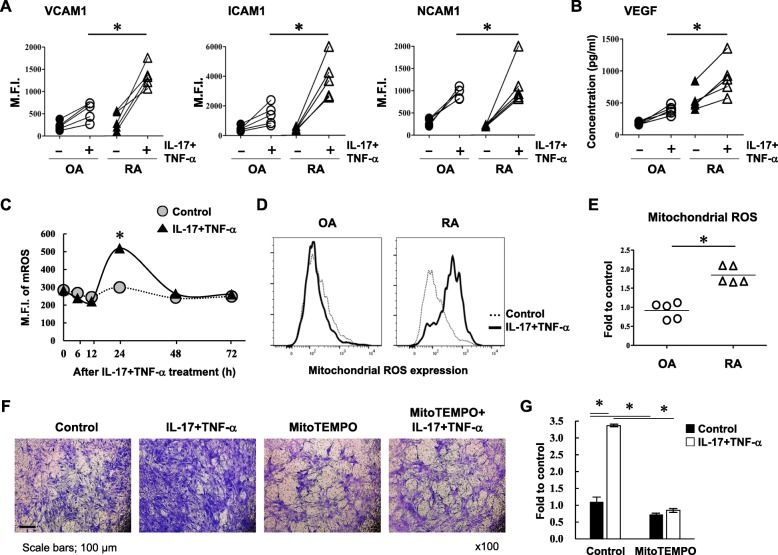
Cytokines preferentially enhance VCAM1, VEGF, and ROS expression in RA FLS compared to OA FLS. RA and OA FLS were stimulated with or without IL-17 (10 ng/ml) and TNF-α (10 ng/ml) for 1 h. Following replacement of culture media, flow cytometry analysis was performed after 23 h with the indicated antibodies. **a** Data represent the mean fluorescence intensity (M.F.I.) for each factor. **b** Levels of secreted VEGF in stimulated and unstimulated RA and OA FLS were measured using an ELISA. **c** Mitochondria-specific ROS (mROS) were detected by MitoSox dye at the indicated time points after cytokine stimulation. Results are presented as mean. **d** The unstimulated control is represented by the dotted line, and cytokine-stimulated cells are represented by the bold line. **e** ROS levels in RA and OA FLS at 24 h are shown. Data are shown as fold change of the M.F.I. in stimulated cells compared to unstimulated control cells. Each symbol represents an individual donor. Each group contains five donors. **f** Following treatment with 20 μM MitoTEMPO for 1 h, RA FLS were stimulated with IL-17 (10 ng/ml) and TNF-α (10 ng/ml) for 1 h. Cell migration was measured using a transwell chamber after 23 h and staining with crystal violet dye. **g** Following solubilization, the crystal violet dye was quantitated. The bar represents the mean. Data represent one experiment, which was performed in triplicate with similar results. *p < 0.05. Scale bar, 100 μm. Magnification is × 100

The joint angiogenic process is mediated by various factors, such as cell adhesion molecules, cytokines/chemokines, and proteases [20]. Adhesion molecules, such as VCAM1, play a critical role in FLS migration and mediate joint inflammation and destruction [21]. We thus determined whether IL-17 and TNF-α promote increased expression of adhesion molecules in RA and OA FLS. Cells were incubated with IL-17 and TNF-α for 1 h, and surface expression of VCAM1, ICAM1, and NCAM1 were analyzed after 23 h. VCAM1, ICAM1, and NCAM1 were increased by nearly 3-fold in OA FLS following cytokine exposure, while expression of these adhesion molecules in RA FLS were significantly increased by 5- to 8-fold following cytokine stimulation (Fig. 2a). The angiogenic growth factor VEGF was also examined in FLS following exposure to IL-17 and TNF-α. Under basal conditions, VEGF exhibited a 3-fold increase in RA FLS compared to OA FLS; however, cytokine stimulation upregulated VEGF similarly in both RA and OA FLS (Fig. 2b).

Increased VCAM1 was mediated by NADPH oxidase-derived ROS and promoted cell migration and angiogenesis [22]. Therefore, we hypothesized that elevated ROS levels may mediate the enhanced migration and invasion observed in RA FLS following cytokine stimulation. Following incubation with IL-17 and TNF-α, the levels of mitochondria-specific ROS in RA FLS were significantly enhanced at 24 h compared to control unstimulated RA FLS (Fig. 2c). There was no difference after 48 h. Next, intracellular ROS expression in RA and OA FLS was analyzed following IL-17 and TNF-α treatment for 24 h. ROS levels were substantially upregulated in RA FLS following cytokine stimulation compared to levels in unstimulated RA FLS controls (1.84 ± 0.20), whereas intracellular ROS levels were not changed in OA FLS following cytokine stimulation (0.91 ± 0.19; Fig. 2d, e). To determine more precisely the role of cytokines in controlling cell migration through mitochondrial specific ROS, we measured cytokine-induced RA FLS migration after treatment with MitoTEMPO mitochondria-targeted antioxidant. IL-17- and TNF-α-mediated cell migration was significantly attenuated by MitoTEMPO pretreatment (Fig. 2f, g). Taken together, these results suggest that a differential increase in ROS induced by cytokines leads to the difference in migration and invasion between RA and OA FLS.

### Overexpression of NOX4 by IL-17 and TNF-α in RA FLS

**Fig. 3:**
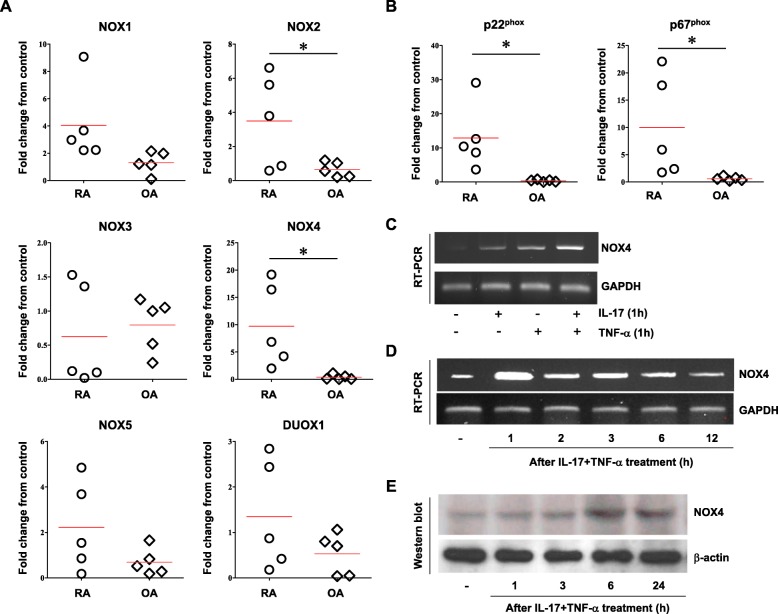
Effects of IL-17 and TNF-α treatment on NOX isoforms in RA and OA FLS. **a**, **b** RA and OA FLS were stimulated with or without IL-17 (10 ng/ml) and TNF-α (10 ng/ml) for 1 h, and then target mRNA levels were assessed by real-time PCR. GAPDH was used as a control. Data represented as fold change compared to control, respectively. Each symbol represents an individual donor. The bar represents the mean. *p < 0.05. **c**, **d** RA FLS were incubated with or without IL-17 (10 ng/ml) and TNF-α (10 ng/ml), and then mRNA expression of *NOX4* was analyzed by RT-PCR at the indicated time points. **e** Protein levels of NOX4 were assessed by western blot at the indicated time points following treatment with IL-17 (10 ng/ml) and TNF-α (10 ng/ml). GAPDH or β-actin was used as loading control. Data represent one experiment, which was performed in triplicate with similar results

ROS generation is closely regulated by the NOX assembly system [23]. To determine whether NOX is involved in the differential expression of ROS in RA and OA, FLS were treated with IL-17 and TNF-α for 1 h and analyzed by real time PCR for NOX family gene expression. Among *NOX* isoforms, *NOX4* and *NOX2* expression was significantly enhanced in RA FLS (Fig. 3a). Expression of the NOX2/4-binding proteins, *p22phox* and *p67phox*, was also highly upregulated in RA FLS following IL-17 and TNF-α treatment (Fig. 3b). The more NOX2, NOX4, p22phos, and p67phos were expressed by cytokines, the higher levels of ROS were produced in individual RA FLS (supplementary data 1). After treatment with IL-17 and/or TNF-α for 1 h, *NOX4* mRNA level was examined. In accordance with cell migration tendency, combination of IL-17 and TNF-α markedly increased *NOX4* expression in RA FLS (Fig. 3c). The increased mRNA expression of *NOX4* peaked 1 h after cytokine stimulation in RA FLS (Fig. 3d). Following incubation of RA FLS with IL-17 and TNF-α, NOX4 protein levels were markedly elevated for 6 h up to 24 h compared to unstimulated control FLS (Fig. 3e). These data showed that *NOX4* may be a major contributor to the enhanced ROS-mediated migration and invasion of FLS in RA.

### Decreased migration and invasion by NOX4 inhibition

**Fig. 4:**
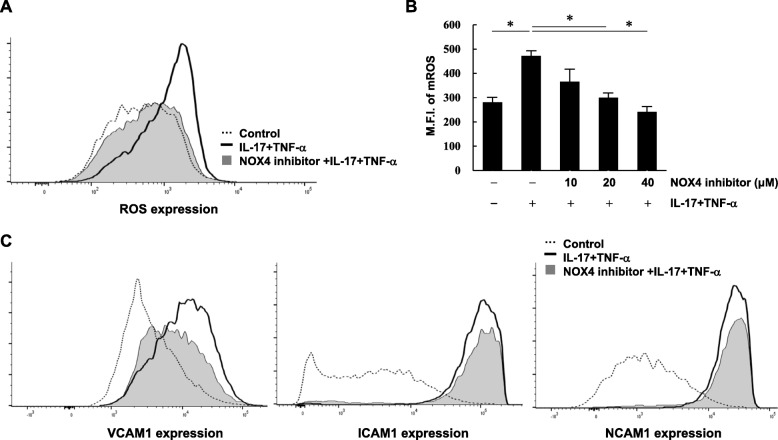
IL-17- and TNF-α-induced ROS and VCAM1 are downregulated by NOX4-specific inhibition in RA FLS. RA FLS were pre-incubated with 40 μM of NOX4 inhibitor GLX351322 for 1 h, and then cells were stimulated with or without IL-17 (10 ng/ml) and TNF-α (10 ng/ml) for 1 h. **a** After 23 h, mitochondria-specific ROS was detected by MitoSox dye. The unstimulated control is shown as a dotted line, and cytokine stimulation is shown as a bold line. The gray-shaded area indicates treatment with both NOX4 inhibitor and cytokines. **b** ROS levels were quantitated in cells incubated with increasing amounts of NOX4 inhibitor and then stimulated with or without IL-17 and TNF-α. Data are shown as the mean fluorescence intensity (M.F.I.) ± S.E.M. *p < 0.05. **c** Flow cytometry analysis was performed using the indicated antibodies. Data represent one experiment, which was performed in triplicate with similar results

**Fig. 5:**
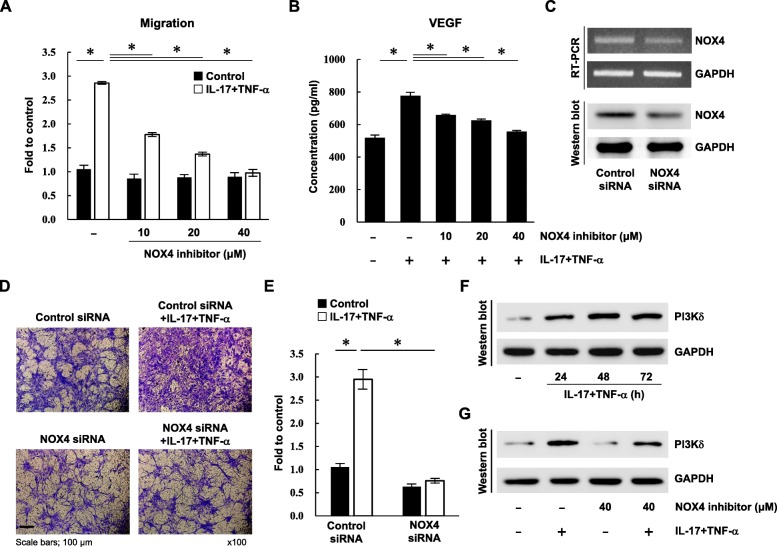
IL-17- and TNF-α-mediated invasion is attenuated by NOX4 siRNA in RA FLS. RA FLS were pre-incubated with 0, 10, 20, and 40 μM NOX4 inhibitor (GLX351322) for 1 h and then stimulated with or without IL-17 (10 ng/ml) and TNF-α (10 ng/ml) for 1 h. **a** Cell migration after 23 h was measured using a transwell chamber. After solubilization, crystal violet dye was measured to determine migration. Data represent a fold change of optical density of crystal violet stained cells compared to unstimulated control cells. **b** Culture supernatants were analyzed by ELISA to measure secreted levels of VEGF. **c** RA FLS were transfected with NOX4 siRNA. After 24 h, *NOX4* levels were assessed by RT-PCR and western blot. **d** Following NOX4 inhibition, cells were stimulated with or without IL-17 (10 ng/ml) and TNF-α (10 ng/ml) for 1 h. Cell invasion was evaluated after 3 days with a Matrigel-coated transwell chamber. Representative transwell chambers with crystal violet-stained cells are shown. Scale bar, 100 μm. Magnification is × 100. **e** After solubilization, crystal violet dye was measured. Data represent a fold change of optical density of crystal violet stained cells compared to control. **f** PI3Kδ activation were assessed in RA FLS at the indicated time points following treatment with IL-17 (10 ng/ml) and TNF-α (10 ng/ml). **g** RA FLS were pre-incubated with 40 μM NOX4 inhibitor (GLX351322) for 1 h and then stimulated with or without IL-17 (10 ng/ml) and TNF-α (10 ng/ml) for 1 h. After 48 h, cells were harvested and measured the expression of PI3Kδ. Data represent one experiment, which was performed in triplicate with similar results. GAPDH was used as a loading control. The bar represents the mean. *p < 0.05

We next wanted to determine whether NOX4 is a critical regulator in IL-17- and TNF-α-induced migration and invasion in RA. RA FLS were incubated with increasing doses of the NOX4-specific inhibitor GLX351322 for 1 h, and then cells were treated with IL-17 and TNF-α for 1 h. After 23 h, the cytokine-induced ROS levels were substantially reduced by the NOX4 inhibitor in a dose-dependent manner (Fig. 4a, b). Among the cytokine-induced adhesion molecules, only VCAM1 was distinctly decreased following NOX4 inhibition (Fig. 4c). Cell migration and VEGF secretion were also significantly reduced by NOX4 inhibition in RA FLS (Fig. 5a, b). When FLS were treated with NOX4-specific inhibitor alone, cell functions were not affected (data not shown).

To confirm the role of NOX4 in cell invasion, we reduced the mRNA levels of *NOX4* in RA FLS using siRNA transfection (Fig. 5c). Following 24 h, NOX4 silencing decreased NOX4 protein by 0.57 fold in western blot analysis. After verification of NOX4 silencing, RA FLS were incubated with IL-17 and TNF-α for 1 h, and cell invasion was assessed using a Matrigel-coated transwell chamber assay. When RA FLS were transfected with control siRNA, IL-17 and TNF-α markedly enhanced the invasion capabilities of RA FLS; however, *NOX4* knockdown suppressed the invasive ability of stimulated FLS compared with those treated with control siRNA (Fig. 5d, e). Despite IL-17 and TNF-α treatment, FLS invasion was inhibited compared to untreated controls in *NOX4*-silenced RA FLS. These findings demonstrate that cytokine-induced migration and invasion are dependent on a NOX4-VCAM1-VEGF pathway.

In the study by Beatrix Bartok et al., phosphoinositide 3-kinase δ (PI3Kδ) played as a critical regulator in migration and invasion of synoviocytes in RA [24]. We hypothesized that PI3Kδ might have a role in NOX4 mediated cell invasion. When RA FLS were stimulated with IL-17 and TNF-α, the protein level of PI3Kδ was markedly increased up to 72 h (Fig. 5f). On the other hand, NOX4 inhibitor GLX351322 attenuated cytokine-mediated PI3Kδ activation (Fig. 5g). These data suggested that PI3Kδ may contribute in NOX4-mediated cell migration and invasion in RA FLS.

## Discussion

FLS are the major population in the synovium and play a critical role in arthritis pathogenesis. In this study, we investigated the effects of the arthritis-associated cytokines IL-17 and TNF-α on the migration and invasion of FLS. IL-17 and TNF-α are the primary focus in RA disease research, and combined blockade of both cytokines has been suggested as a novel therapy for patients who are unresponsive to selective TNF-α inhibition [25, 26]. Here, we revealed that NOX4 mediated a differential response to IL-17 and TNF-α stimulation in RA FLS compared to OA FLS. Specifically, *NOX4* was increased by cytokine exposure and was required for RA FLS migration and invasion via increased expression of VCAM1 and VEGF. Our findings therefore highlight NOX4 as a potential treatment target in RA.

Interestingly, while RA FLS demonstrated a rapid and robust increase in migration and invasion following cytokine stimulation, FLS from OA donors showed little or no enhancement. The differential responses may be related to the different amounts of IL-17 and TNF-α in synovium. Indeed, IL-17 was detected at higher concentrations in RA synovium than in OA or control synovium [27, 28]. Although migration and invasion in OA FLS did not change with cytokine stimulation for 1 h, expression of VCAM1, ICAM1, NCAM1, and VEGF was increased 2–3-fold. Therefore, we concluded that the adhesion molecules and angiogenic factors were differentially regulated by these cytokines through a mechanism that is distinct from cell migration and invasion.

Next, we investigated whether IL-17 and TNF-α affected ROS levels in RA and OA FLS, because ROS have been shown to directly induce cell migration and invasion in the context of cancer [29]. Mitochondria-specific ROS expression was increased by cytokines in RA FLS but not OA FLS, and similar results were found for invasiveness. Our findings strongly suggest that IL-17 and TNF-α induce a differential increase in ROS that leads to the distinct invasion capabilities between RA and OA.

The increased intracellular ROS in FLS may negatively influence RA-associated synovium changes, such as immune cell activation and pro-inflammatory cytokine secretion, as well as invasion of FLS into bone [30, 31]. In relation to cell migration, ROS-generating enzymes in the NOX family have been suggested as therapeutic target molecules in various diseases, such as cardiovascular disease, autoimmune disease, and inflammation [32, 33]. It was shown that RA FLS exhibited aggressive features, such as hypoxia, invasion, and inflammation, which are similar to cancer [34]. NOX4 overexpression in human colorectal cancer was associated with poor prognosis and increased tumor migration and invasion [35]. In vascular inflammatory disease, *NOX4* knockdown mediated a significant decrease in inflammation [36, 37]. Although inhibition of excessive ROS has previously been suggested as an important target in RA treatment, few studies have investigated the role of NOX and RA FLS.

When mRNA expression in RA FLS was analyzed in response to IL-17 and TNF-α stimulation, *NOX4* was increased most among the NOX family members. Following NOX4 inhibition in RA FLS, cytokine-induced ROS, VCAM1, VEGF, migration, and invasion were downregulated to levels observed for untreated controls. Together, these results indicate that IL-17- and TNF-α-mediated *NOX4* expression activates a ROS-VCAM1-VEGF pathway that contributes to FLS migration and invasion in RA.

One limitation of this study is the use of ex vivo human FLS, which may not precisely reflect the in vivo response. Further study using an animal RA model is needed. In addition, the mRNA expression of *NOX2* was also significantly increased in RA FLS, and therefore, additional study of the role of *NOX2* in RA is warranted.

In conclusion, we revealed that NOX4 was upregulated in RA FLS following IL-17 and TNF-α stimulation, leading to aggressive migration and invasion via a ROS-VCAM1-VEGF pathway. These findings suggest that NOX4 may be a critical factor in RA pathogenesis and that this enzyme may provide a therapeutic target for treatment of RA and other inflammatory diseases.

## Conclusions

We investigated the effects of IL-17 and TNF-α on migration and invasion of FLS isolated from patients with RA and OA and further explored the effects of NOX4 inhibition in the pathogenesis of RA. Here, we revealed that NOX4 mediated a differential response to IL-17 and TNF-α stimulation in RA FLS compared to OA FLS. Specifically, *NOX4* was increased by cytokine exposure and was required for RA FLS migration and invasion via increased expression of VCAM1 and VEGF. Our findings therefore highlight NOX4 as a potential treatment target in RA.

## Supplementary information

**Additional file 1: Supplementary data 1.** Correlative expressions among NOX isoforms in IL-17 and TNF-α stimulated RA FLS. RA FLS were stimulated with or without IL-17 (10 ng/ml) and TNF-α (10 ng/ml) for 1 h, and then target mRNA levels were assessed by real-time PCR. GAPDH was used as a control. Data represented as fold change compared to control, respectively. Each symbol represents an individual donor.

## Data Availability

The datasets used and analyzed during the current study are available from the corresponding author on reasonable request.

## Abbreviations

DAS28: 28-Joint Disease Activity Score
FITC: Fluorescein isothiocyanate
FLS: Fibroblast-like synoviocytes
HC: Healthy control subjects
ICAM1: Intercellular adhesion molecule 1
IL-17: Interleukin 17
MFI: Mean fluorescence intensity
NADPH: Nicotinamide adenine dinucleotide phosphate
NCAM1: Neural cell adhesion molecule 1
NOX: Nicotinamide adenine dinucleotide phosphate oxidase
OA: Osteoarthritis
PBMC: Peripheral blood mononuclear cell
PE-PC5: Phycoerythrin-cyanine 5
PI3K δ: Phosphoinositide 3-kinase δ
RA: Rheumatoid arthritis
ROS: Reactive oxygen species
TNF-α: Tumor necrosis factor alpha
VCAM1: Vascular cell adhesion molecule 1
VEGF: Vascular endothelial growth factor
