# Supplementary figures and images for: The effect of nicotinamide adenine dinucleotide phosphate oxidase 4 on migration and invasion of fibroblast-like synoviocytes in rheumatoid arthritis
Source: Arthritis Res Ther. 2020 May 15;22:116. doi: 10.1186/s13075-020-02204-0 (PMC7227051; doi:10.1186/s13075-020-02204-0)

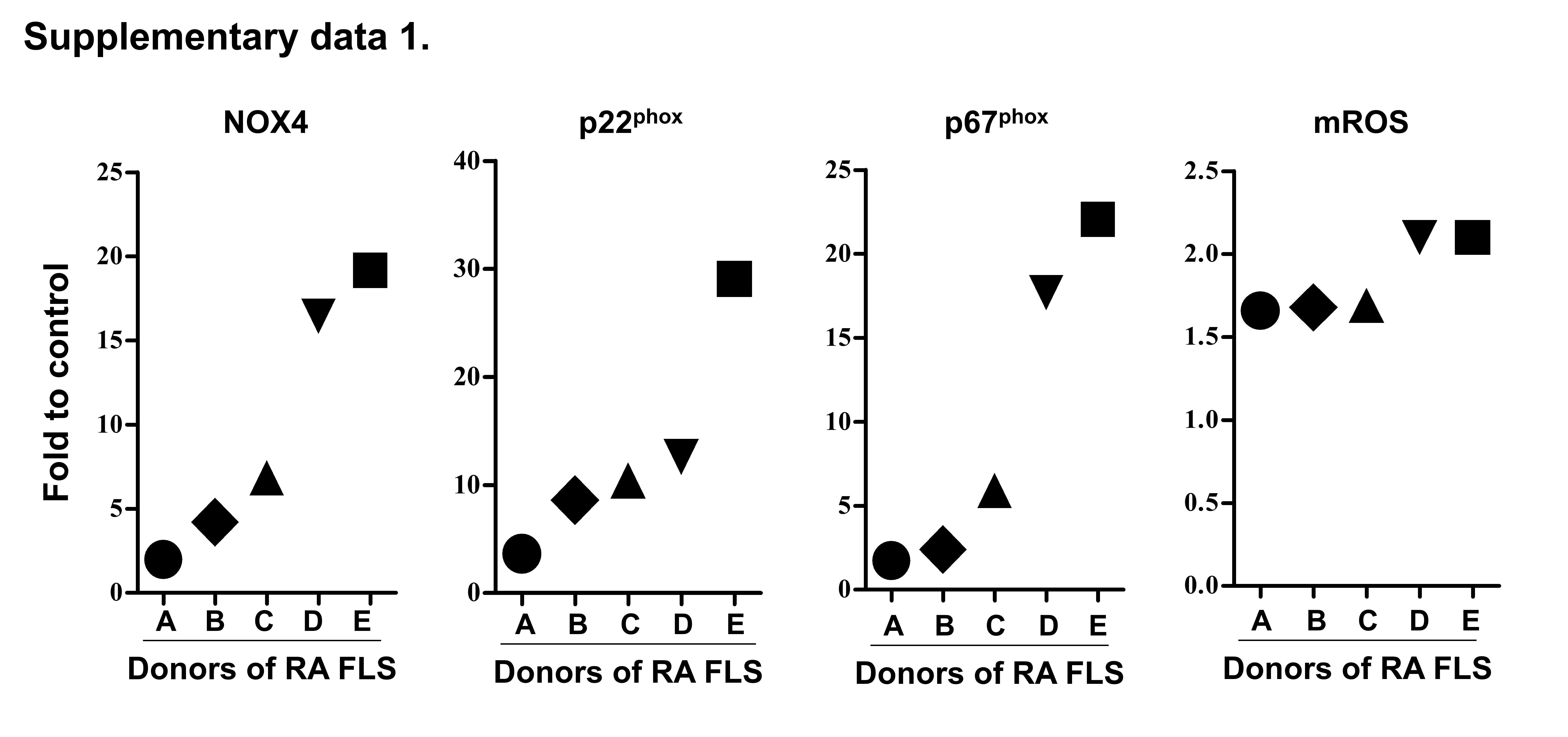

Supplement: Supplementary file 1 — Additional file 1: Supplementary data 1. Correlative expressions among NOX isoforms in IL-17 and TNF-α stimulated RA FLS. RA FLS were stimulated with or without IL-17 (10 ng/ml) and TNF-α (10 ng/ml) for 1 h, and then target mRNA levels were assessed by real-time PCR. GAPDH was used as a control. Data represented as fold change compared to control, respectively. Each symbol represents an individual donor. [file 13075_2020_2204_MOESM1_ESM.tif]
